# Application of Machine Learning Algorithms in Plant Breeding: Predicting Yield From Hyperspectral Reflectance in Soybean

**DOI:** 10.3389/fpls.2020.624273

**Published:** 2021-01-12

**Authors:** Mohsen Yoosefzadeh-Najafabadi, Hugh J. Earl, Dan Tulpan, John Sulik, Milad Eskandari

**Affiliations:** ^1^Department of Plant Agriculture, University of Guelph, Guelph, ON, Canada; ^2^Department of Animal Biosciences, University of Guelph, Guelph, ON, Canada

**Keywords:** artificial intelligence, data-driven model, ensemble methods, high-throughput phenotyping, random forest, recursive feature elimination

## Abstract

Recent substantial advances in high-throughput field phenotyping have provided plant breeders with affordable and efficient tools for evaluating a large number of genotypes for important agronomic traits at early growth stages. Nevertheless, the implementation of large datasets generated by high-throughput phenotyping tools such as hyperspectral reflectance in cultivar development programs is still challenging due to the essential need for intensive knowledge in computational and statistical analyses. In this study, the robustness of three common machine learning (ML) algorithms, multilayer perceptron (MLP), support vector machine (SVM), and random forest (RF), were evaluated for predicting soybean (*Glycine max*) seed yield using hyperspectral reflectance. For this aim, the hyperspectral reflectance data for the whole spectra ranged from 395 to 1005 nm, which were collected at the R4 and R5 growth stages on 250 soybean genotypes grown in four environments. The recursive feature elimination (RFE) approach was performed to reduce the dimensionality of the hyperspectral reflectance data and select variables with the largest importance values. The results indicated that R5 is more informative stage for measuring hyperspectral reflectance to predict seed yields. The 395 nm reflectance band was also identified as the high ranked band in predicting the soybean seed yield. By considering either full or selected variables as the input variables, the ML algorithms were evaluated individually and combined-version using the ensemble–stacking (E–S) method to predict the soybean yield. The RF algorithm had the highest performance with a value of 84% yield classification accuracy among all the individual tested algorithms. Therefore, by selecting RF as the metaClassifier for E–S method, the prediction accuracy increased to 0.93, using all variables, and 0.87, using selected variables showing the success of using E–S as one of the ensemble techniques. This study demonstrated that soybean breeders could implement E–S algorithm using either the full or selected spectra reflectance to select the high-yielding soybean genotypes, among a large number of genotypes, at early growth stages.

## Introduction

The world population is projected to exceed nine billion individuals by 2050, which will require significant improvements in the yield of major crops that contribute to global food security (Tilman et al., 2009; Foley et al., 2011; Alexandratos and Bruinsma, 2012; Dubey et al., 2019). Increasing the yield is the primary goal of most plant breeding programs for major crops, such as soybean (*Glycine max*), which is the world’s most widely grown leguminous crop and an important source of protein and oil for food and feed (Hartman et al., 2011). In the area of plant breeding, however, measuring primary traits, such as yield, which is under influenced by a combination of quantitative and qualitative traits, in large breeding populations consisting of several thousand genotypes is time and labor-consuming (Araus and Cairns, 2014; Cai et al., 2016; Xiong et al., 2018). Breeding for yield is known as a highly complex and non-linear process due to the genetic and environmental factors (Collins et al., 2008). Therefore, breeding approaches that are established based on secondary traits (e.g., yield component traits and reflectance bands), which are strongly correlated with the primary trait, enable plant breeders to efficiently recognize promising lines at early growth stages (Ma et al., 2001; Jin et al., 2010; Montesinos-López et al., 2017).

The combination of high-throughput genotyping and phenotyping technologies have enabled plant breeders to make their early growth stage selections more accurate while it reduced the evaluation time and cost in their breeding programs (Rutkoski et al., 2016). Although there has been significant progress in high-throughput genotyping in recent years with a direct impact on current plant breeding challenges (Araus and Cairns, 2014; Tardieu et al., 2017; Araus et al., 2018), acquisition of high-throughput field phenotyping is still a bottleneck in most breeding programs (Furbank and Tester, 2011; Araus et al., 2018).

Remote sensing of spectral reflectance is considered as an efficient high-throughput phenotyping tool (Araus and Cairns, 2014; Tardieu et al., 2017), which aims to measure the spectral reflectance efficiently at several plant growth and development stages in large breeding populations (Rutkoski et al., 2016). It is well documented that the spectral properties are genotype-specific and influenced by the anatomy, morphology, and physiology of plants (Kycko et al., 2018; Schweiger et al., 2018) and, therefore, can be used for screening plant genotypes with different agronomic potentials.

Analyzing large datasets consisting of spectral reflectance data requires intensive computational and statistical analyses, which is still challenging in many plant breeding programs (Lopez-Cruz et al., 2020). Nowadays, machine learning algorithms have drawn attention from researchers to develop model-based breeding methods that can improve the efficiency of breeding processes (Hesami et al., 2020a). Recently, one of the most common artificial neural networks (ANNs), the multilayer perceptron (MLP) developed by Pal and Mitra (1992), has been broadly used for modeling and predicting complex traits, such as yield, in different breeding programs (Geetha, 2020). MLP can be considered as a non-linear computational method employed for various tasks such as classification and regression of complex systems (Chen and Wang, 2020; Hesami et al., 2020b). This algorithm is able to detect the connection and relationship between the input and output (target) variables and quantify the inherent knowledge existing in the datasets (Ghorbani et al., 2016; Hesami et al., 2020b). This algorithm includes several highly interconnected processing neurons that can be used in parallel to detect a solution for a specific problem (Ghorbani et al., 2016; Geetha, 2020). Support vector machines (SVMs), developed by Vapnik (2000), are known as one of the powerful and easy to use machine learning algorithms that can recognize patterns and behavior of non-linear relationships (Auria and Moro, 2008; Su et al., 2017). Some of the advantages of SVMs over MLP are linked to the complexity of the networks. SVMs usually use a large number of learning problem formulations leading to solving a quadratic optimization problem (Feng et al., 2020; Hesami et al., 2020b). In theory, SVM has to be better performance because of using structural risk minimization inductive principles rather than the empirical risk minimization inductive principle (Belayneh et al., 2014). In addition to MLP and SVM, random forest (RF) (Breiman, 2001) is another method for data modeling with a computational efficient training phase and very high generalization accuracy. RF has been broadly used in areas such as object recognition (Lepetit et al., 2005), skin detection (Khan et al., 2010), plant phenomics (Falk et al., 2020), and genomics (Mokry et al., 2013).

Machine learning algorithms are subject to overfitting, mainly because of limited training data and dependent on single predictive models (Ali et al., 2014; Feng et al., 2020). Ensemble techniques, in which a group of algorithms are exploited to combine all the possible predictions for the ultimate prediction used to address this shortage (Dietterich, 2000). By using ensemble models, the predictive performances were improved for yield prediction in Alfalfa (Feng et al., 2020), Nicosia wastewater treatment plant (Nourani et al., 2018), and plant lncRNAs (Simopoulos et al., 2018). Boosting, bagging, and stacking are three of the most commonly used ensemble models (Dietterich, 2000; Feng et al., 2020). The bagging method was first introduced by Breiman (1996) as a variance reduction approach for different algorithms such as decision trees or other algorithms that employed variable selection and fitting in a linear model (Galar et al., 2011). Boosting algorithms have been introduced by Schapire (1999) to serve as the alternative for the bagging method (Drucker and Cortes, 1996). Unlike bagging methods, which are parallel ensemble techniques, boosting methods are known as sequential ensemble techniques of base models by exploiting the dependencies of each algorithm (Dietterich, 2000; Feng et al., 2020). Many studies reported the successfulness of using bagging-RF and stochastic gradient boosting in predicting the yield of different crops (Pal, 2007; Gandhi et al., 2016; Aghighi et al., 2018; Zhang Z. et al., 2019). Bagging and boosting ensemble techniques commonly combine homogeneous algorithms for interpretation, while stacking methods tend to use heterogeneous algorithms and adjust the difference between them to increase precision (Dietterich, 2000; Zhou, 2012; Feng et al., 2020).

The successful use of machine learning algorithms for predicting the performance of different agronomic traits, including yield, are reported in Alfalfa (Feng et al., 2020), *Senecio* species (Carvalho et al., 2013), grassland (Feilhauer et al., 2017; Rocha et al., 2018), and chrysanthemum (Hesami et al., 2019). However, the application of machine learning algorithms for predicting soybean yield from hyperspectral reflectance data is still unexplored and required comprehensive studies. Ensemble-based methods have successfully been applied to improve the prediction accuracies in artificial intelligence and computer vision (Ali et al., 2014; Feng et al., 2018, 2020; Ju et al., 2018) and, therefore, they may improve the accuracy of the yield prediction in this study. Thus, the main objectives of this study are: (1) to investigate the potential use of hyperspectral reflectance for predicting soybean yield, (2) to identify appropriate time-point of soybean growth stages for collecting hyperspectral reflectance to maximize yield prediction accuracy, and (3) to have a comparative study of individual and ensemble machine learning algorithms to improve the accuracy of predicting yield. The results of this study might help soybean breeders to increase the efficiency of selecting superior lines by estimating the final yield at early growth stages using spectral reflectance combined with machine learning approaches.

## Materials and Methods

### Experimental Locations and Plant Materials

**FIGURE 1:**
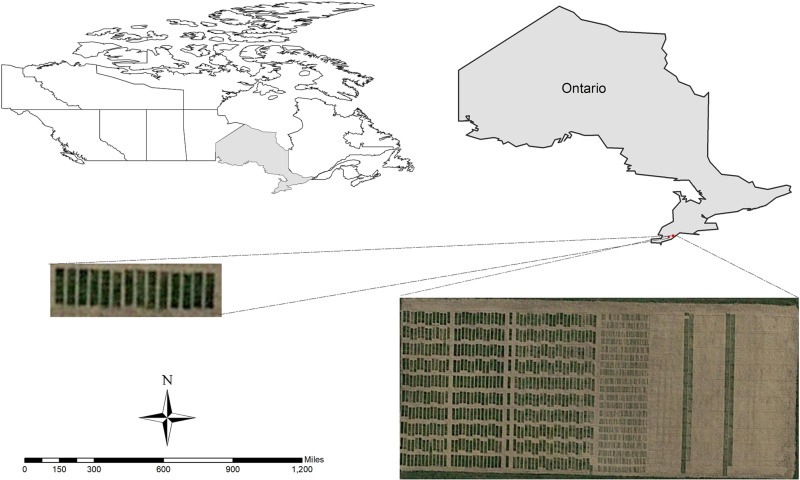
The location of the experiments in 2018 and 2019.

The research was conducted at the University of Guelph, Ridgetown Campus, in 2018 and 2019. A panel of 250 soybean genotypes was grown under field condition at two locations: Ridgetown (42°27′14.8″N 81°52′48.0″W, 200 m above sea level) and Palmyra (42°25′50.1″N 81°45′06.9″W, 195 m above sea level), in Ontario, Canada, during two consecutive growing seasons in 2018 and 2019 (Figure 1).

The soybean genotypes used in this study were the core germplasms of the soybean breeding program at the University of Guelph, Ridgetown, that have been collected in the past 35 years and used for genetic studies and cultivar developments. The experiments were conducted using randomized complete block designs (RCBD) with two replications in four environments (two locations × two years). Overall, there were 500 soybean plots per environment and 1000 soybean plots per year. In order to reduce the possible spatial variability in the field, each experiment was analyzed by nearest-neighbor analysis (NNA) as one of the error control strategies by using double covariate analysis (Stroup and Mulitze, 1991; Bowley, 1999; Katsileros et al., 2015). Each plot consisted of five rows, each 4.2 m long with a row spacing of 43 cm. The seeding rate was 57 seeds/m^2^. At the end of the season, the three inside rows were machine harvested for estimating total seed yields (Ton ha^–1^).

### Phenotypic Evaluations

#### Yield Collection

Soybean seed yield (Ton ha^–1^) was measured using three out of five harvested rows for each plot and adjusted to a 13% moisture level. The best linear unbiased prediction (BLUP) as a mixed model was used to calculate the average seed yield production for each soybean genotype across different environments (Goldberger, 1962).

#### Hyperspectral Reflectance Data Collection

In this study, the focus was on the spectral reflectance bands that are typically classified as the visible (VIS) and near-infrared (NIR) spectral components (Albetis et al., 2017). Canopy hyperspectral reflectance measurements were collected during the soybean growth and development stages at R4, where pods are 1.91 cm long at one of the four uppermost nodes, and R5, where seeds are 0.31 cm long in pods at one of the four uppermost nodes (Pedersen et al., 2004).

Each soybean genotype’s hyperspectral reflectance properties were collected using a UniSpec-DC Spectral Analysis System (PP Systems International, Inc., 110 Haverhill Road, Suite 301 Amesbury, MA, United States). The machine covers 250 reflectance bands between 350 nm and 1,100 nm with a bandwidth of 3 nm. The field-of-view of the sensor was approximately 25° and covered a sample area of 0.25 m^2^. Dark reference was used for calibrating the dual channels, and Spectralon panels were used to characterize incoming solar radiation. For each plot, three measurements were recorded, and their average, calculated by the BLUP model, was used as the reflectance band datapoint. All of the measurements were performed as close to solar noon as possible—the data for each stage were collected in 1 day, from 11:00 AM to 2:00 PM, to minimize the signal noise associated with the environment.

### Data Pre-processing and Statistical Analyses

The existence of noise during hyperspectral reflectance measurement is inevitable, typically caused by sensors and electronic fluctuations (Ozaki et al., 2006). Therefore, it would be critical to have a pre-processing step for the collected data in order to increase the accuracy of the study. The hyperspectral data and yield of 250 soybean genotypes were pre-processed using the R software (version 3.6.1) to remove potential noises that randomly occur across the whole spectra resulting in misinterpretations. After checking the quality of reflectance bands and detecting outliers by using principal component analysis (PCA) for each genotype, 245 genotypes were selected for further analyses (Serneels and Verdonck, 2008). As a result of sensor-specific artifacts, reflectance bands at the two edges of the hyperspectral reflectance spectrum, 350–395 nm and 1,005–1,100 nm, were removed from the original data. The collected contiguous hyperspectral reflectance data was also reduced from 395 to 1005 nm with a 3 nm interval to a 10 nm interval leading to a total of 62 variables. Data scaling and centering were applied in order to improve reflectance properties in the pre-processing and the pre-treatment steps (Rossel, 2008). For each reflectance band variable, the Savitzky–Golay filter was applied for improving the signal-to-noise ratio (Savitzky and Golay, 1964).

**FIGURE 2:**
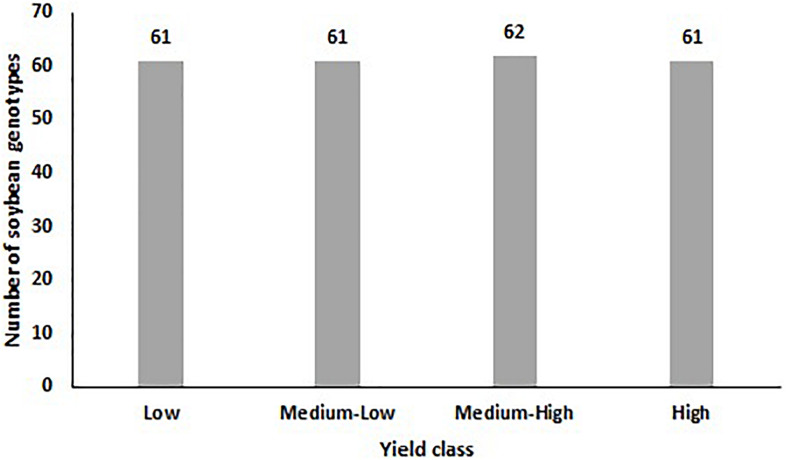
The distribution of soybean genotypes in each yield class.

As shown in Figure 2, the measured soybean yield was divided into four classes with equal numbers (∼) of data points in ascending order: Low (0–24.99% of total yield), medium-low (25–49.99% of total yield), medium-high (50–74.99% of total yield), and high (75–100% of total yield) yield. While 62 reflectance bands were considered as input variables, the classified yield was chosen as the output variable.

### Variable Selection

Feature or variable selection is usually applied before developing the machine learning algorithms for reducing the data dimensionality, specifically in the small training datasets. One of the common approaches for variable selection is the recursive feature elimination (RFE) approach, which is easy to configure and effectively select the most relevant variables that predict the output (Chen and Jeong, 2007). Therefore, the RFE was run to indicate the initial variable importance scores and eliminate the reflectance band variables with the lowest importance score. Afterward, the process was recursively repeated until the ranking was indicated for all the reflectance bands. The package *caret* (Kuhn, 2008) in R software version 3.6.1 was used for running RFE.

### Data-Driven Modeling

**FIGURE 3:**
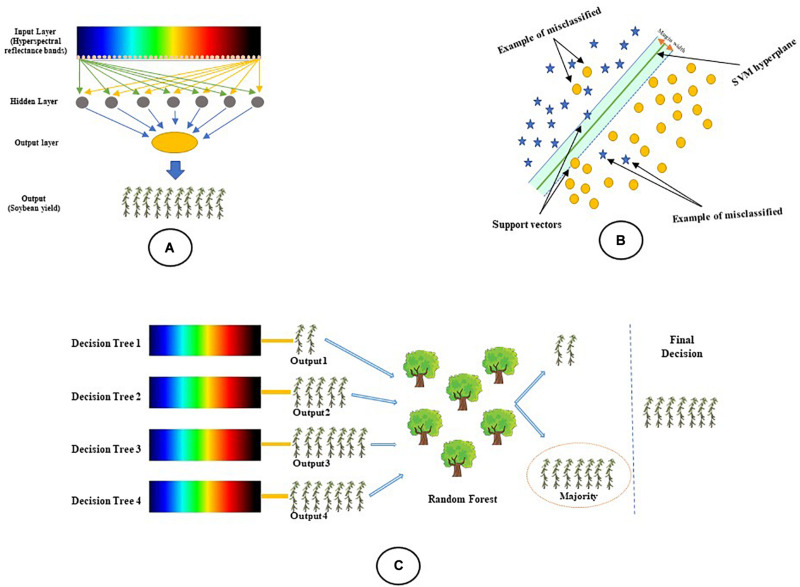
A schematic representation of the machine learning algorithms used in this study to classify the soybean yield using reflectance bands: **(A)** Multilayer perceptron, **(B)** Support vector machine, and **(C)** Random forest.

Three of the most commonly used algorithms in the literature, multilayer perceptron (MLP), the support vector machine (SVM), and random forest (RF) (Filippi and Jensen, 2006; Chen et al., 2007; De Castro et al., 2012; Makantasis et al., 2015; Zhang N. et al., 2019; Šestak et al., 2019), were chosen and used for predicting the soybean yield. Figure 3A shows the MLP algorithm including an input layer, one or more hidden layers, and an output layer of completely interconnected neurons. Each neuron unit produces an output based on a sigmoid function derived from a linear combination of outputs from a previous layer (Wang et al., 2009). SVM (Figure 3B) is a set of related supervised learning methods that can recognize patterns used for classification analyses (Suykens and Vandewalle, 1999; Shao et al., 2012). The objective of SVM is to use hyperplanes for determining the optimal separation of yield classes. The random forest (RF) approach generates a series of trees representing a subset *n* of independent observations (Figure 3C). A detailed description of these machine learning algorithms can be found in Taillardat et al. (2016) and Meinshausen (2006). All of the relevant parameters in each machine learning algorithm were optimized based on the input variables.

We employed an ensemble method based on a stacking strategy (E–S) to improve the prediction performance. The results from individual algorithms were collected and combined together via the stacking procedure described in Dietterich (2000), where an algorithm with the highest accuracy performance was selected as the metaClassifier for this ensemble model. The Weka software version 3.9.4 (Hall et al., 2009) was used for running all machine learning algorithms and the ensemble method.

### Quantification of Machine Learning Performance

**FIGURE 4:**
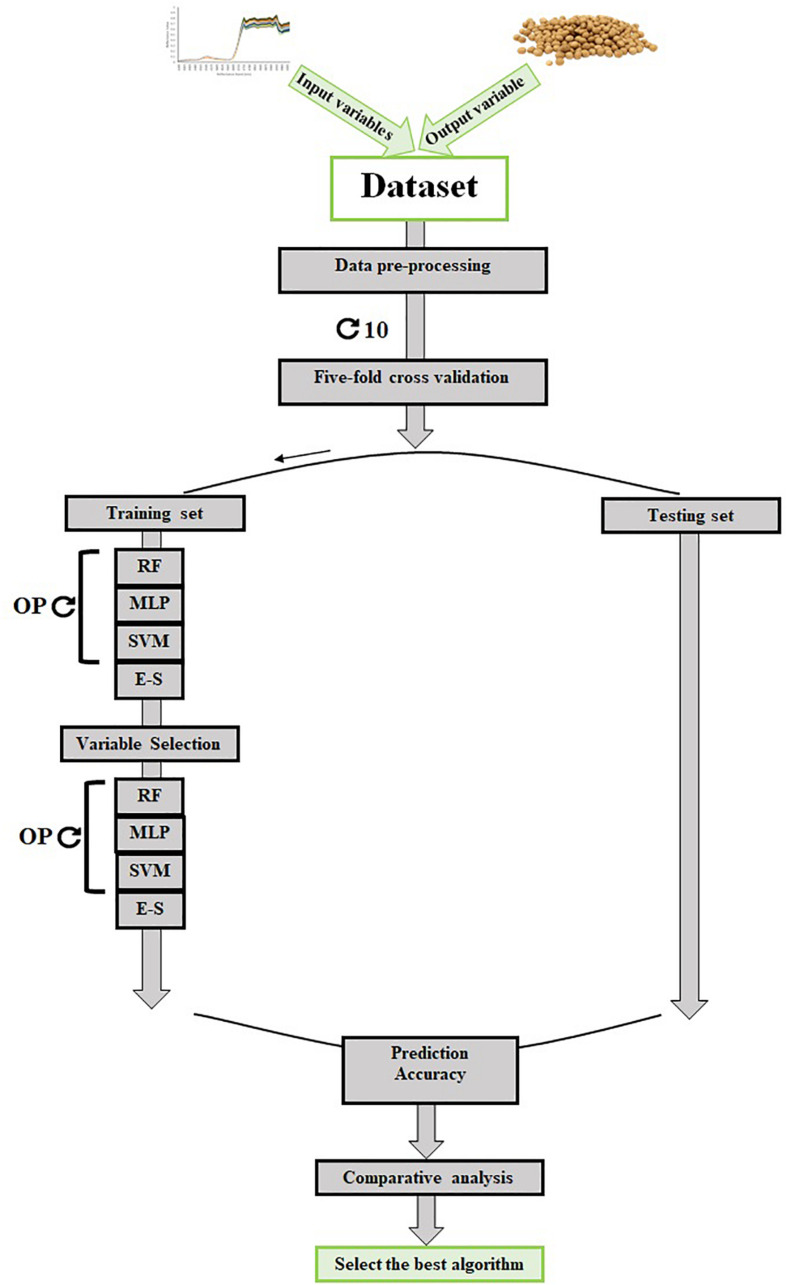
The scheme of data collection and machine learning algorithm development and validation. OP, optimizing parameters; MLP, multilayer perceptron; SVM, support vector machine; RF, random forest; E–S, ensemble–stacking strategy.

In this study, the fivefold cross-validation strategy (Siegmann and Jarmer, 2015) with 10 repetitions was used to measure the classification quality of all the tested ML algorithms (Figure 4). In order to evaluate each algorithm, the values of precision (Eq. 1), recall (Eq. 2) as a measure of sensitivity, F-measure (Eq. 3), and Matthews correlation coefficient (MCC, Eq. 4) for validation dataset were measured using the following formulas:

(1) $$\text{Precision}=\frac{TP}{TP+FP}$$

(2) $$\text{Recall}=\frac{TP}{TP+FN}$$

(3) $$\text{F-measure}=2\times \frac{\text{Precision}\times \text{Recall}}{\text{Precision}+\text{Recall}}$$

(4) $$\text{MCC}=\frac{TP\times TN-FP\times FN}{\sqrt{\left(TP+FP\right)\left(TP+FN\right)\left(TN+FP\right)\left(TN+FN\right)}}$$

where *TP* stands for the number of true positives, *TN* is the number of true negatives, *FP* stands for the number of false positives, and *FN* is the number of false negatives.

### Visualizing and Analyzing

The Microsoft Excel software (2016), *ggplot2* (Wickham, 2011), and *ggvis* (Dennis, 2016) packages in the R software version 3.6.1 were used to conduct statistical analyses and visualize the results.

## Results

### Yield Statistics and Spectral Profiles

**FIGURE 5:**
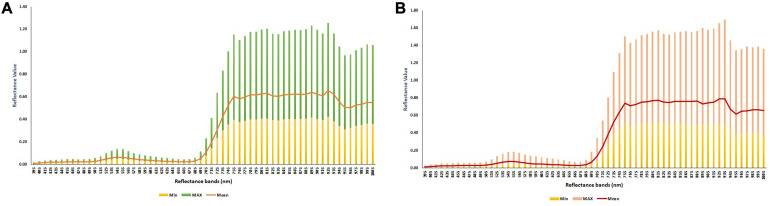
The minimum, mean, and maximum values of each reflectance band were measured for 245 soybean genotypes evaluated at **(A)** R4 and **(B)** R5 growth stages at four different field environments.

In the current study, the average yield of 245 soybean genotypes, evaluated in four environments, ranged from 2.58 to 5.71 ton ha^–1^ with a mean and standard deviation of 4.22 and 0.57 ton ha^–1^, respectively. The minimum, mean, and maximum values of each reflectance bands evaluated for all the genotypes across the four environments at the R4 and R5 growth stages are reported in Figure 5. At both growth stages, while the reflectance values showed small ranges of variation among the genotypes between 395 and 695 nm, the bands greater than 705 nm showed large variations within the population.

### Variable Selection

**FIGURE 6:**
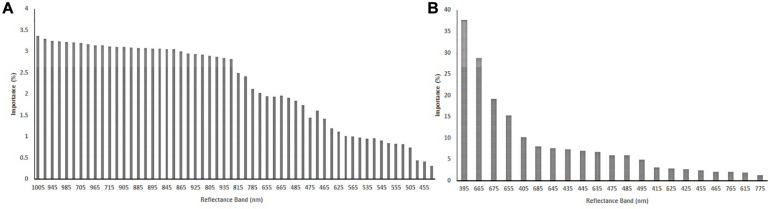
The importance of selected variables based on the recursive feature elimination (RFE) strategy for soybean reflectance bands measured at R4 **(A)** and R5 **(B)** soybean growth stages.

**FIGURE 7:**
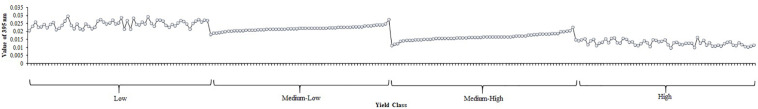
The soybean yield classes versus the 395 nm reflectance band at R5 growth stages.

The importance values of all 62 reflectance band variables for predicting yield were estimated using the RFE strategy for the R4 (Table 1) and R5 (Table 2) growth stages. For the R4 growth stage, the 1005 nm and the 605 nm bands had the highest and lowest importance values (%) for classifying the soybean yield, respectively. Based on RFE analysis, 56 of the reflectance bands were selected for training the algorithm, as shown in Figure 6A. At the R5 growth stage, the highest and lowest importance values (%) were found at 395 nm and the 725 nm bands, respectively. Out of 62 reflectance bands, 21 reflectance bands were selected to train the algorithms based on RFE strategy, which were considered selected variables (-VS) for further analyses. Among the 21 selected reflectance bands, three bands were in the violet, six in the blue, two in the green, eight in the red, and two were in the near-infrared (NIR) regions of the spectrum (Figure 6B). By using RFE for the R4 growth stage dataset, the top five high importance reflectance bands were located in the violet and NIR regions of the spectra. However, for R5, the violet and red regions had the top five high importance reflectance bands (Tables 1, 2). The violet region, specifically the 395 nm band, had the highest importance values in both growth stages. The plotting of the soybean yield versus reflectance values at 395 nm (R5 growth stage) illustrated that the values for 395 nm in the high yielding class ranged from 0.009 to 0.016 which lower than values for the low yielding class, ranged from 0.020 to 0.029 (Figure 7). The difference between the reflectance values of high and low yielding classes was statistically significant at the significance level of 0.05 (data were not shown). Among all the tested bands, the 395 nm band measured at R5 was considered as the best solo reflectance band for discriminating soybeans for their yield potential.

**TABLE 1:** Reflectance band ranking using the recursive feature elimination (RFE) strategy at R4 soybean growth stage.

Reflectance band (nm)	Ranking	Reflectance band (nm)	Ranking
1005	1	775	32
395	2	655	33
945	3	675	34
755	4	665	35
985	5	825	36
995	6	485	37
705	7	695	38
745	8	475	39
965	9	615	40
955	10	465	41
715	11	515	42
975	12	625	43
905	13	685	44
725	14	565	45
885	15	575	46
875	16	535	47
895	17	645	48
765	18	545	49
845	19	635	50
915	20	555	51
865	21	415	52
735	22	505	53
925	23	595	54
855	24	455	55
805	25	585	56
795	26	445	57
935	27	425	58
835	28	435	59
815	29	525	60
405	30	495	61
785	31	605	62

**TABLE 2:** Reflectance band ranking using the recursive feature elimination (RFE) strategy at R5 soybean growth stage.

Reflectance band (nm)	Ranking	Reflectance band (nm)	Ranking
395	1	965	32
665	2	845	33
675	3	865	34
655	4	905	35
405	5	915	36
685	6	515	37
645	7	695	38
435	8	885	39
445	9	875	40
635	10	895	41
475	11	585	42
485	12	925	43
495	13	935	44
415	14	575	45
625	15	955	46
425	16	715	47
455	17	755	48
465	18	535	49
765	19	975	50
615	20	555	51
775	21	985	52
795	22	995	53
815	23	525	54
805	24	545	55
785	25	565	56
505	26	945	57
605	27	705	58
825	28	1005	59
855	29	745	60
595	30	735	61
835	31	725	62

### Growth Stage Comparison

**FIGURE 8:**
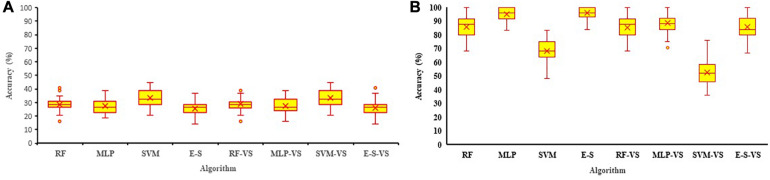
The accuracy of RF, MLP, SVM, and E–S algorithms for predicting soybean yield using full and RFE selected variables (-VS) measured at R4 **(A)** and R5 **(B)** soybean growth stages in four environments. The mean performance was shown as × in each figure. MLP, multilayer perceptron; SVM, support vector machine; RF, random forest; E–S, ensemble–stacking strategy; RFE, recursive feature elimination.

In order to investigate which of the growth stages is better for collecting reflectance data and predicting the soybean yield, the reflectance bands collected at each soybean growth stage were analyzed using the three machine learning algorithms. The average classification accuracy for validation dataset ranged between 12 and 43% using the R4 data and between 12 and 99% using the R5 data, which indicated that the R5 soybean growth stage is, in general, a better stage for collecting reflectance data if the goal is to predict the yield (Figure 8). Therefore, R5 was selected for further machine learning algorithm analyses. The results of individual and ensemble machine learning algorithms using R4 data are available in Figure 8 and Supplementary Figure 1.

### Comparative Analysis of the Developed Models

**FIGURE 9:**
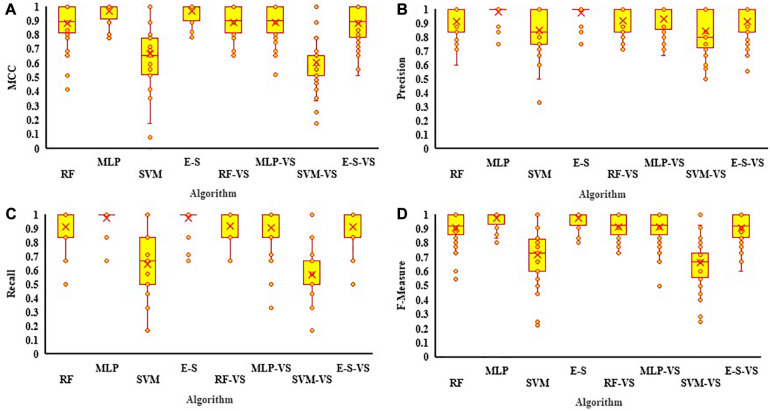
The estimate values of **(A)** Matthews correlation coefficient (MCC), **(B)** Precision, **(C)** Recall, and **(D)** F-Measure for RF, MLP, SVM, and the E–S algorithms used for predicting soybean yield from all and selected variables collected (-VS) at the R5 growth stage. The mean performance is indicated with × in each figure. MLP, multilayer perceptron; SVM, support vector machine; RF, random forest; E–S, ensemble–stacking strategy; RFE, recursive feature elimination.

All three algorithms (RF, MLP, and SVM), as well as the E–S model, were trained using both full (62 bands) and selected (21 bands) variables at R5, and the summaries of the confusion matrices were presented in Supplementary Table 1. Regarding the comparative analyses of individual algorithms using all variables, RF, MLP, and SVM had the highest to lowest MCC values equal to 0.84, 0.76, and 0.66, respectively (Figure 9A). For the selected variables, the MCC values for RF and MLP declined to 0.80 and 0.73, respectively, while the value for SVM slightly increased to 0.73. The E–S method outperformed all the individual algorithms obtaining an MCC value of 0.93, using all variables, and 0.87, using selected variables (Figure 9A). In general, among all the individual tested algorithms, the RF algorithm had the highest performance with the values of 84 and 80% yield classification accuracy using all variables and selected variables, respectively.

When using full variables, the precision values for RF, MLP, and SVM were 0.91, 0.83, and 0.82, respectively. However, by using selected variables, the precision values for RF and MLP declined to 0.87 and 0.78, respectively, while the SVM performance was improved (0.87) when compared against all variables (Figure 9B). The E–S model had a precision of 0.96 using all variables and 0.90 using selected variables. Using all variables, the highest recall value was obtained for RF with a value of 0.84, followed by MLP and SVM with the values of 0.83 and 0.68, respectively. However, the recall value of SVM increased to 0.72 using selected variables. The recall values of RF and MLP declined when selected variables were used (Figure 9C). E–S had the highest recall values, with 0.94 and 0.90 for full and selected variables, respectively.

To have a better interpretation of precision and recall values, the F-measure was evaluated for each and every algorithm. Using all variables, the F-measures of RF, MLP, and SVM were estimated to be 0.87, 0.81, and 0.71, respectively (Figure 9D). F-measure values were decreased for RF (0.84) and MLP (0.80) using selected variables. However, for SVM algorithm, the F-measure value was increased to 0.77 using selected variables. The E–S algorithm overperformed all the individual machine learning algorithms by having an F-measure value of 0.94, using all variables, and 0.90, using selected variables.

## Discussion

One of the objectives of this study was to find the best growth stage for collecting reflectance data suitable for predicting soybean yields. In this study, the hyperspectral reflectance data were collected at the reproductive stages of R4 and R5, in which pods and seeds are developed. R4 and R5 are known as critical growth and development stages in soybean since stresses can impose significant impacts on the yield at these stages and, therefore, soybean genotypes with different levels of tolerance to stresses can be discriminated from one another at these stages (Sweeney et al., 2003). For example, the results of a study by Eck (1987) showed that imposing soybeans to water deficit stress during the R1 to R3 growth stages reduce seed yields up to 9-13%. However, imposing the same soybeans to water deficit stress during R4 to R5 reduced seed yields up to 46%. Water deficit stress can less influence the total seed yield when occurring anytime beyond the R5 growth and development stage. Therefore, measuring hyperspectral reflectance at R4 and R5 would be more informative for predicting the soybean yield classes since the final yield production has been to some extent established at these two stages for all the genotypes. Our results indicated that R5 is a better stage to measure reflectance bands for predicting the yield. Ma et al. (1996) reported significant correlations between leaf photosynthetic rates and leaf greenness at R4 and R5, while this correlation was not significant at R6. In Soybean, the leaf photosynthetic rate can be changed significantly during the growth stages that, in turn, can empower different genotypes to recover the yield losses caused by temporary environmental stresses (Ferris et al., 1998; Siebers et al., 2015). Studies showed that environmental stresses at R5 can damage the soybean yield greater than that at R4 (Fehr et al., 1981) since the plants have less time to recover for yield before physiological maturity. It can be hypothesized that predicting yield of genotypes with different genetic potential by using reflectance bands that are measured at R5 is more reliable since the final yield productions have already been established, to some extent, for all the genotypes. The current study showed that the reflectance bands collected at R5 are more reliable and informative for predicting yield than the data collected at R4.

Several studies reported the strong correlation between reflectance bands and yield in different crop plants such as alfalfa (Kayad et al., 2016; Feng et al., 2020), wheat (Prey and Schmidhalter, 2019), maize (Lane et al., 2020), rice (Wan et al., 2020), and sugarcane (Verma et al., 2020). The visible reflectance bands can be splitted into three main regions, red (650–700 nm), green (495–570 nm), and violet–blue (390–495 nm) (Hennessy et al., 2020). Most studies were emphasized the importance of red spectral bands or the combined use of red and red edge bands as one solid index in predicting the total yield (Jolly et al., 2005; Filippa et al., 2018; Lykhovyd, 2020; Phan et al., 2020; Tiwari and Shukla, 2020). In this study, we identified highly ranked bands in the violet and red regions for classifying the soybean seed yield, centered at 395 nm, 665 nm, and 675 nm (Table 2). The violet and red spectral regions can be associated with the absorption of plant pigments such as carotenoid, anthocyanins, and chlorophyll (Merzlyak et al., 2003; Richter et al., 2016; Hennessy et al., 2020). Carotenoid plays a pivotal role in discrimination of senescent leaves (Richter et al., 2016; Hennessy et al., 2020). The importance of 395 nm band in soybean yield prediction at R5 growth stage can refer to the fact that soybean at R5 growth stage initiates the senescence and decay of chlorophyll resulting in better discrimination of the genotypes with different photosystems functioning and photoprotection capabilities. However, there is no report on the solid effect of the 395 nm reflectance band in the physiological process of soybean or any other plants.

In order to have accurate yield prediction and avoid model overfitting, machine learning algorithms may benefit from using a variable selection process to reduce the dimensionality of the data to an appropriate level (Hennessy et al., 2020). Existing variable selection methods can be categorized based on their applications, complexities, and accuracy (Zheng et al., 2020). One of the most commonly used variable selection methods is the RFE approach that provides an acceptable performance with moderate computational exertions (Guyon et al., 2002; Granitto et al., 2006). The successful use of RFE to reduce the number of input variables has been reported in many studies (Granitto et al., 2006; Chen and Jeong, 2007; Feng et al., 2020). The efficiency of using selected variables for predicting classified soybean yield over full reflectance band variables was evaluated using the RFE method. Using RFE method might decrease the value of the parameters such as precision, recall, MCC, and F-measure to avoid overfitting (Loughrey and Cunningham, 2004). This is a small price to pay, especially if the decrease in performance is not significant. Among all the tested individual machine learning algorithms, RF had the highest performance using either full or selected variables. This high performance may come from the nature of the RF algorithm, in which trees are trained using multiple random subsamples of the original dataset. This feature gives RF this ability to generate better and more stable predictions for new instances not necessarily included in the training dataset (Liaw and Wiener, 2002).

Multilayer perceptron was another machine learning algorithm that was exploited in this study. MLP was previously applied in different areas such as weed science (Tamouridou et al., 2017) or drought tolerance (Etminan et al., 2019), but not in soybean for yield prediction. This study found MLP to be the second-best machine learning algorithm for predicting the soybean yield. Previous studies reported a high likelihood of overfitting for neural network algorithms (Lawrence and Giles, 2000; Murakoshi, 2005). For MLP, common parameters such as the number of hidden layers, the number of neurons in each layer, or training time can be used to control overfitting; however, the degree of overfitting would vary throughout the input variables (Lawrence and Giles, 2000).

Support vector machine is also one of the most common machine learning algorithms that have been broadly used in different areas such as plant tissue culture (Hesami and Jones, 2020), image classification (Lin et al., 2011), genes classification (Duan et al., 2005), and drug disambiguation (Björne et al., 2013). SVM is usually used when scientists have to deal with large numbers of features and high sparsity (Nguyen and De la Torre, 2010). Although the prediction accuracy of the SVM algorithm was lower than the values for RF and MLP in this study, its performance was slightly increased when the selected variables were used. An increase in SVM performance using selected variables was also reported in previous studies (Su and Yang, 2008; Tan et al., 2010; Alirezanejad et al., 2020). It might be due to this fact that selecting relevant variables can improve the performance of SVM through ameliorating its feature interpretability, computational efficiency, and generalization performance (Nguyen and De la Torre, 2010; Roy et al., 2015).

In order to see we can improve the prediction accuracy in this study through the combined use of the machine learning algorithms, RF, MLP, and SVM were used in constructing E–S, and RF was chosen as the metaClassifier for this ensemble algorithm. By using the E–S approach, we improved the prediction accuracy using either full or selected variables. A successful use of the E–S method has recently been reported for predicting the yield in alfalfa (Feng et al., 2020). When using the E–S approach, it is necessary to include self-sufficient, independent, and diverse ML algorithms in the analyses (Araya et al., 2017; Feng et al., 2020), which have a minimum dependency from one another and sufficient powers to predict the dependent variable, soybean yield classes in this study (Araya et al., 2017; Feng et al., 2020). The above criteria are important to be considered when individual ML algorithms are selected to combine in a given E–S analysis. In this study, RF, MLP, and SVM are selected as individual algorithms to be used in the E–S analyses because of their independent prediction methods as well as having different training approaches. By using the E–S approach, the prediction accuracy increased to 0.93, using all variables, and to 0.87, using selected variables, showing the success of using E–S as one of the ensemble techniques.

## Conclusion

Pre-harvest soybean yield classifications and estimations are important for grain policy-making and food security across worldwide. Spectral reflectance is considered as an efficient phenotyping tool that can help breeders to make their selections at lower cost at a fast pace. The objectives of this study were to demonstrate the best soybean growth stage for measuring hyperspectral reflectance and evaluating the three most commonly used ML algorithms along with introducing the E–S method in predicting the soybean yield using reflectance variables. Soybean R5 growth stage was identified as the better stage than R4 for measuring hyperspectral reflectance. In addition to using 62 reflectance bands as the full variables, the RFE method was used to reduce the dimensionality of the data, and therefore, 21 most important bands were selected as the selected reflectance variables. Using both full and selected reflectance variables, RF overperformed all individual algorithms. Therefore, RF was selected as the metaClassifier for E–S. E–S had the highest prediction accuracy as one of the ensembles combined approaches compared to an individual ML algorithm. Therefore, E–S was recommended as a reliable and appropriate ML algorithm for predicting the soybean yield using reflectance variables. This study provides an applicable pipeline for using hyperspectral reflectance data and suitable ML algorithms for the development of high yielding soybeans, which can be used in large soybean breeding programs for selecting high-yielding soybeans at pre-harvesting stages. The developed methodology in this study can open a reliable and new window in using spectral reflectance for selecting high yielding genotypes in different crops.

## Data Availability Statement

The raw data supporting the conclusions of this article will be made available by the authors, without undue reservation.

## Author Contributions

MY-N performed the experiments, modeled, summed up, and wrote the manuscript. HE, DT, and JS revised the manuscript. ME designed and lead the experiments and revised the manuscript. All authors have read and approved the final manuscript.

## Conflict of Interest

The authors declare that the research was conducted in the absence of any commercial or financial relationships that could be construed as a potential conflict of interest.
