# Supplementary material for: Application of Machine Learning Algorithms in Plant Breeding: Predicting Yield From Hyperspectral Reflectance in Soybean
Source: Front Plant Sci. 2021 Jan 12;11:624273. doi: 10.3389/fpls.2020.624273 (PMC7835636; doi:10.3389/fpls.2020.624273)
Supplement: Supplementary file 1 [file Table_1.DOCX]

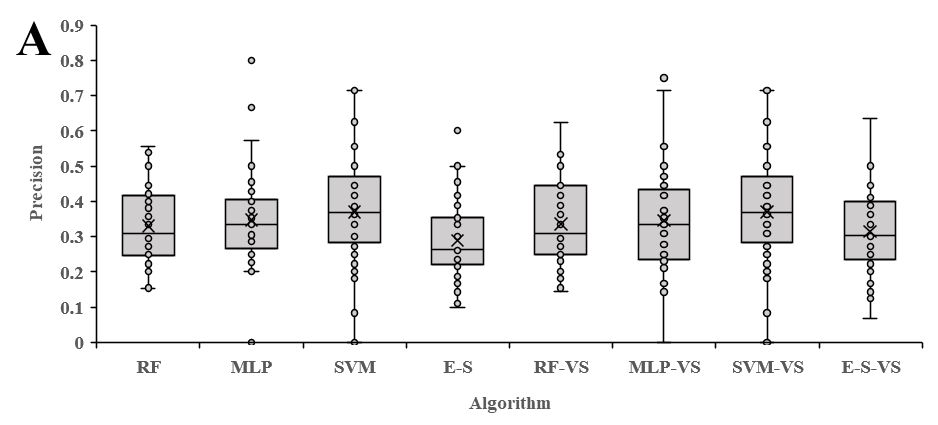

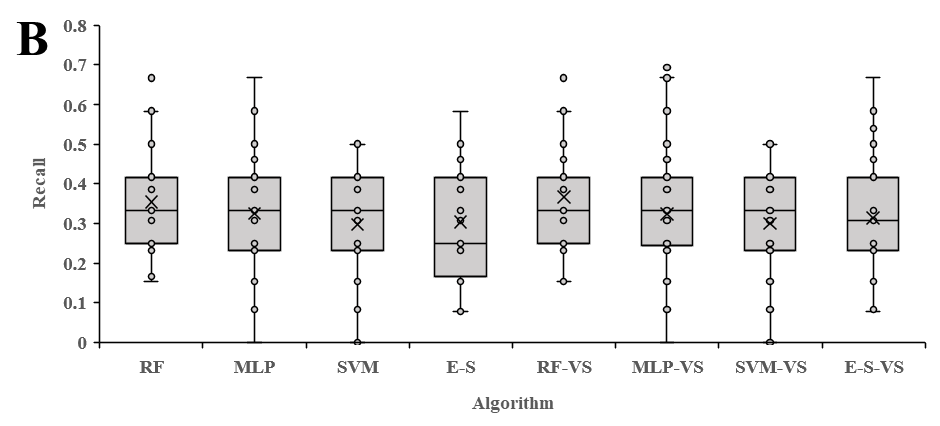


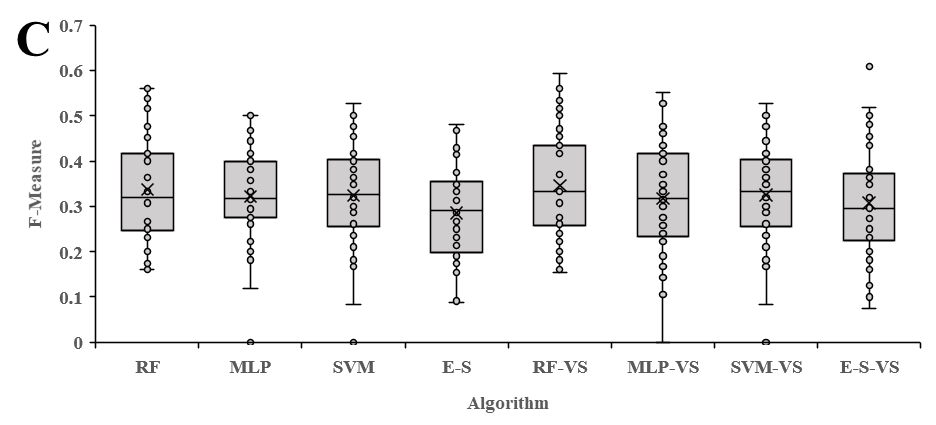

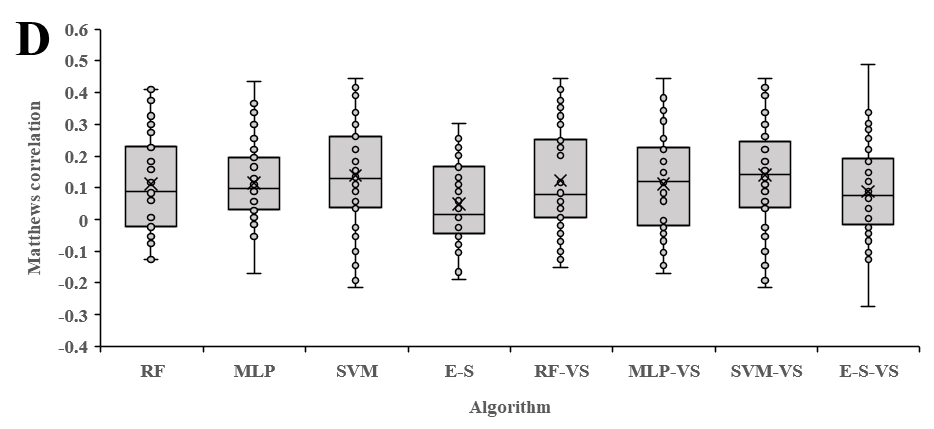


**Figure S1**. Performance and error evaluation of RF, MLP, SVM and E-S model in soybean yield prediction using full and selected reflectance bands from R4 growth stage (VS: variable selection).

| **Table S1.** Confusion matrix based on the performance of RF, MLP, SVM, and Ensemble-Stacking model in predicting the soybean yield using full and selected variables in R5 soybean growth stage. | | | | | | | | | | | |
| --- | --- | --- | --- | --- | --- | --- | --- | --- | --- | --- | --- |
| **Full Variable** | | | | | | **Selected Variable** | | | | | |
| **Algorithm** |  | **Low** | **Medium-Low** | **Medium-High** | **High** | **Algorithm** |  | **Low** | **Medium-Low** | **Medium-High** | **High** |
| **RF** | **Low** | 58 | 3 | 0 | 0 | **RF** | **Low** | 57 | 4 | 0 | 0 |
|  | **Medium-Low** | 6 | 53 | 2 | 0 |  | **Medium-Low** | 5 | 54 | 2 | 0 |
|  | **Medium-High** | 0 | 3 | 47 | 12 |  | **Medium-High** | 0 | 3 | 46 | 13 |
|  | **High** | 0 | 0 | 5 | 56 |  | **High** | 0 | 0 | 9 | 52 |
|  |  |  |  |  |  |  |  |  |  |  |  |
| **MLP** | **Low** | 54 | 7 | 0 | 0 | **MLP** | **Low** | 52 | 9 | 0 | 0 |
|  | **Medium-Low** | 2 | 56 | 3 | 0 |  | **Medium-Low** | 5 | 55 | 1 | 0 |
|  | **Medium-High** | 0 | 3 | 56 | 3 |  | **Medium-High** | 0 | 2 | 54 | 6 |
|  | **High** | 0 | 0 | 6 | 55 |  | **High** | 0 | 0 | 7 | 54 |
|  |  |  |  |  |  |  |  |  |  |  |  |
| **SVM** | **Low** | 33 | 24 | 4 | 1 | **SVM** | **Low** | 37 | 21 | 2 | 0 |
|  | **Medium-Low** | 7 | 39 | 15 | 0 |  | **Medium-Low** | 7 | 40 | 14 | 0 |
|  | **Medium-High** | 0 | 13 | 37 | 12 |  | **Medium-High** | 0 | 10 | 38 | 14 |
|  | **High** | 0 | 2 | 15 | 44 |  | **High** | 0 | 2 | 14 | 47 |
|  |  |  |  |  |  |  |  |  |  |  |  |
| **E-S** | **Low** | 57 | 4 | 0 | 0 | **E-S** | **Low** | 53 | 8 | 0 | 0 |
|  | **Medium-Low** | 4 | 57 | 0 | 0 |  | **Medium-Low** | 7 | 49 | 5 | 0 |
|  | **Medium-High** | 0 | 0 | 54 | 8 |  | **Medium-High** | 0 | 5 | 51 | 6 |
|  | **High** | 0 | 0 | 6 | 55 |  | **High** | 0 | 0 | 8 | 53 |
